# Interoperable Platform to Report Polymerase Chain Reaction SARS-CoV-2 Tests From Laboratories to the Chilean Government: Development and Implementation Study

**DOI:** 10.2196/25149

**Published:** 2021-01-20

**Authors:** Sergio Guinez-Molinos, José María Andrade, Alejandro Medina Negrete, Sonia Espinoza Vidal, Elvis Rios

**Affiliations:** 1 Laboratory of Biomedical Informatics School of Medicine Universidad de Talca Talca Chile; 2 Interoperability Area National Center for Health Information Systems Talca Chile

**Keywords:** COVID-19, SARS-CoV-2, interoperability, laboratory information system, HL7 FHIR, PCR

## Abstract

**Background:**

Testing, traceability, and isolation actions are a central strategy defined by the World Health Organization to contain the COVID-19 pandemic. In this sense, the countries have had difficulties in counting the number of people infected with SARS-CoV-2. Errors in reporting results are a common factor, as well as the lack of interoperability between laboratories and governments. Approaches aimed at sending spreadsheets via email expose patients’ privacy and have increased the probability of errors due to retyping, which generates a delay in the notification of results.

**Objective:**

This study aims to design and develop an interoperable platform to report polymerase chain reaction (PCR) SARS-CoV-2 tests from laboratories to the Chilean government.

**Methods:**

The methodology to design and develop the interoperable platform was comprised of six well-structured stages: (1) creation of a minimum data set for PCR SARS-CoV-2 tests, (2) modeling processes and end points where institutions interchange information, (3) standards and interoperability design, (4) software development, (5) software testing, and (6) software implementation.

**Results:**

The interoperable Fast Healthcare Interoperability Resources (FHIR) platform to report PCR SARS-CoV-2 tests from laboratories to the Chilean government was successfully implemented. The platform was designed, developed, tested, and implemented following a structured methodology. The platform’s performance to 1000 requests resulted in a response time of 240 milliseconds, throughput of 28.3 requests per second, and process management time of 131 milliseconds. The security was assured through a private network exclusive to the Ministry of Health to ensure confidentiality and integrity. The authorization and authentication of laboratories were implemented with a JavaScript Object Notation Web Token. All the PCR SARS-CoV-2 tests were accessible through an application programming interface gateway with valid credentials and the right access control list.

**Conclusions:**

The platform was implemented and is currently being used by UC Christus Laboratory. The platform is secure. It was tested adequately for confidentiality, secure authorization, authentication, and message integrity. This platform simplifies the reporting of PCR SARS-CoV-2 tests and reduces the time and probability of mistakes in counting positive cases. The interoperable solution with FHIR is working successfully and is open for the community, laboratories, and any institution that needs to report PCR SARS-CoV-2 tests.

## Introduction

The COVID-19 pandemic has caused an unprecedented public health crisis. When this issue occurs, technology can effectively support institutions by facilitating the immediate widespread distribution of information in real time [1,2]. To contain the pandemic, the World Health Organization (WHO) defined the testing, traceability, and isolation (TTI) actions [3], considering an early diagnosis as a critical stage. The polymerase chain reaction (PCR)–based tests are effective for diagnostic testing that looks for the SARS-CoV-2 virus’s genetic material, which causes COVID-19 [4].﻿ ﻿As per the Centers for Disease Control and Prevention recommendations [4], the PCR test is the gold standard and accurate method for detecting, tracking, and studying COVID-19 [5].

The COVID-19 pandemic is especially challenging for laboratories tasked with rapid and reliable testing of an increased number of PCR tests [6]. For these tasks, the Laboratory Information Systems (LIS) is fundamental for systematizing this process [7,8]. It avoids low-quality reports and decreases test outcomes’ misdiagnosis, negatively affecting patient administration [8]. One of the most important considerations for the laboratory is maintaining patient data privacy and avoiding mistakes with the information [9]. Vecellio et al [7] determined that approximately 8.1% of handwritten request forms received at the serology laboratory were incorrectly entered into the LIS; a further 2.6% of test request forms had errors not associated with data entry. Overall, 10.7% of all handwritten request forms were affected by one or more errors.

Chile has strengthened its testing capacity by creating a national network of diagnostic laboratories that includes more than 100 authorized centers in the country [10]. Of these, 40 are in public hospitals, more than 32 are in private laboratories, and 28 are in universities, all with heterogeneous technologies for storing and sharing results. They process more than 22,000 PCR tests daily, exceeding 5.6 million tests analyzed nationwide to date. The public sector processes 45% of these tests, the private sector processes 40%, and the remaining 15% is equivalent to the universities’ contribution [10].

On the other hand, the government implemented a web platform to receive the PCR results. The process of transferring these results is still by spreadsheets via email, which is, in most cases, entered manually due to the lack of interoperability between health information systems.

Despite the effort, Chile did not account for 31,412 patients who were infected due to the lack of interoperability between systems (laboratory and government) and using spreadsheets and email as a formal mechanism for notifying PCR test results [11]. Thus, this carries the risk that the data could be modified or errors may occur in their handling. In the United Kingdom, 16,000 cases of COVID-19 were missed from official counting attributable to the use of spreadsheets for sharing results [12]. Furthermore, in Brazil, numerous deaths related to COVID-19 were possibly recorded by mistake due to reporting errors [13]. There are various reasons and errors in the reporting of results. However, a common factor is the lack of interoperability between health information systems.

During the COVID-19 situation, modern health care systems significantly depend on teamwork and communication. The meaningful information exchange within laboratories is needed to provide information when and where required, facilitate quicker and more effective decision making, reduce repeated work, and improve safety with fewer errors [14]. By definition, interoperability is the ability of two or more health information systems to exchange data and use them adequately [14].

In Chile, the interoperability between health care information systems has been difficult. The public and private institutions have not established consensus in the absence of a government strategy that regulates the interoperability’s policies in the health sector. The lack of defined standards and terminologies and a public-private health system regulatory framework have blocked an interconnected health network from functioning.

In this context, the National Center of Health Information Systems (CENS, acronym in Spanish), in collaboration with private and public laboratories, signaled the need to advance to the national strategy of interoperability. This alliance’s focus was connecting laboratories and the government with standards, which would solve the problems associated with health care systems’ interoperability. This contribution was centered on visualizing the need to share information through standards and demonstrating the feasibility and associated benefits with a specific use case.

For reporting PCR SARS-CoV-2 tests from laboratories and to advance a proposal for interoperability between health information systems, this paper proposes a platform designed and developed by CENS with a focus on safely expediting the delivery of the PCR SARS-CoV-2 tests from different laboratories to the Chilean government, avoiding data entry mistakes. We intend to accelerate the emerging interoperability agenda, presenting tangible and transferable results to the national and international community. The platform proposed a solution designed and developed with the international standards set by Health Level 7 (HL7) Fast Healthcare Interoperability Resources (FHIR) [15], enabling the system to be used in a scalable and reusable way for any LIS.

FHIR is an extensive international standard of interoperability that uses lightweight and modern web principles [16]. Compared with other document-centric standards, FHIR takes a modular and scalable approach by exposing the health data entities as services using http-based representational state transfer (REST) [14] and application programming interfaces (APIs). Furthermore, FHIR is more comfortable to implement, as it uses an API-based approach and a choice of JavaScript Object Notation (JSON) or XML for representing the data [17]. We used HAPI FHIR libraries [18] as a complete implementation of the FHIR standard for health care interoperability in Java, providing an opportunity to add existing health care applications’ capabilities.

## Methods

### Overview

The methodology to design and develop the interoperable platform had six well-structured stages (Textbox 1): (1) creation of a minimum data set to report the PCR SARS-CoV-2 tests, (2) modeling processes and end points where institutions interchange information, (3) standards and interoperability design of the FHIR, (4) software development, (5) software testing, and (6) software implementation.

**Methodology to design and develop interoperable solutions. Six well-defined stages with a focus on standardized methods to build an interoperable health care solution.**

**Interoperability design**

- Creation of minimum data set
  Build the minimum data set to build the polymerase chain reaction SARS-CoV-2 report
- Modeling process
  Designs the model of the laboratory process for sharing data with the Chilean government
- Standards and interoperability design
  Matches with the minimum data set and Fast Healthcare Interoperability Resources (FHIR)

**Software developing**

- Software development
  Designs and develops the software solution using the HAPI FHIR libraries to create end points, resources, and messages
- Software testing
  Tests of the functional and nonfunctional requirements for the interoperable platform
- Software implementation
  Pilot software implementation by considering all the documentation and sharing real data between institutions

This methodology was proposed to develop interoperability projects. The methodology complements any methodology for developing software. Three initial phases (first level: interoperability design) were established focusing on data, processes, and standards. The first phase was the creation (and consensus) of a minimum data set to be exchanged. Phase two modeled and formalized the process, which considers obtaining the data and detecting the exchange points. With these inputs, it is now possible to select the appropriate standard and match it (whether it be messaging, resources, or documents) with the HL7 standards. This first level shows the importance of good design for interoperability with the data, process, and standards previous to developing software [14].

The three final phases (second level: software developing) were oriented to include methodologies for developing software, considering developing, testing, and implementing stages. In this development, the platform was created using ﻿agile software methods that support the incremental and iterative approaches for developing robust and interoperable health care information systems [19].

To create the interoperable platform, two full-time computer engineers and the CENS interoperability area leader worked on the platform. They took 2 months to develop the prototype (July to August 2020) and 1 month to make modifications during the pilot application (September). In October 2020, the pilot application was implemented for one laboratory (UC Christus) that sends PCR SARS-CoV-2 results to the local department of health services (Servicio de Salud Metropolitano Sur Oriente).

### Creation of Minimum Data Set

**Figure 1:**
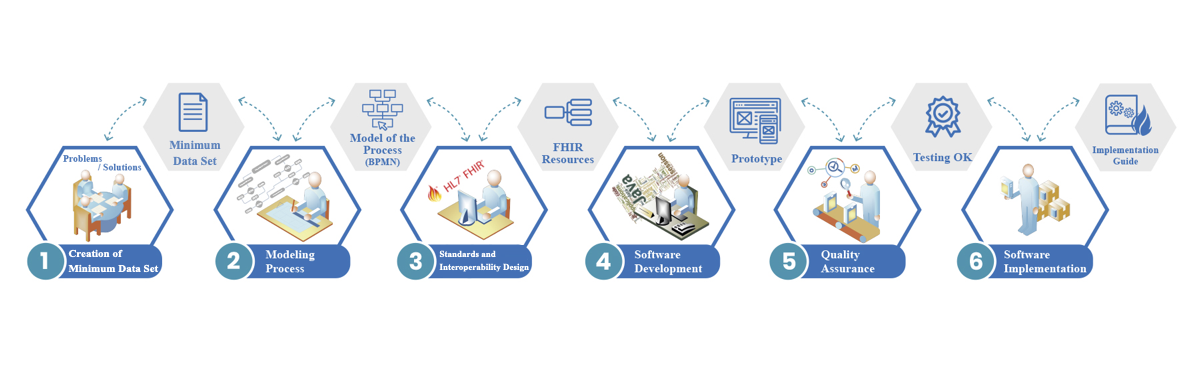
Methodology to design and develop an interoperable software solution. Six well-defined stages with its products and standards involved. BPMN: Business Process Model and Notation; FHIR: Fast Healthcare Interoperability Resources; HL7: Health Level 7.

The first stage to communicate the results between laboratories and the Chilean government was constructing a minimum data set to share the information (Figure 1). The collaboration was essential for the meeting, discussing, and consenting to fulfill the minimum data set needed from the PCR SARS-CoV-2 tests to track and develop scalable and interoperable solutions. In this sense, we met with most of the laboratories that process PCR tests for COVID-19 in Chile. In three online sessions, the institutions discussed the importance of streamlining data and the report’s fields. This group designed and documented the name, data type, cardinality, and possible extensions of the fields. 

### Modeling Process

The modeling of the process is an essential component for interoperable development [14]. It is necessary to understand the process and end points where information between the institutions is interchanged. We used the Business Process Model and Notation (BPMN) [20,21] standard using the free software Camunda Modeler (Camunda Services GmbH) [22]. The diagrams were kept in a web-shared directory and were shared by Cawemo (Camunda Services GmbH) [23], a web application focused on the collaborative editing of BPMN diagrams. This software allows for the creation of projects with the possibility of commenting on elements, exporting and importing, and sharing them with other users differentiated by roles, enabling real-time monitoring and visualization of the latest version of the diagram.

### Standards and Interoperability Design

Once the minimum data set was defined, the process modeled, and the end points detected, the following stage adopted standards and interoperability design. In this phase, we worked with FHIR Release 4 [16]. HL7 developed FHIR as an interoperability standard designed explicitly for web-based exchange and improved its capabilities with emerging web standards such as REST interfaces [14]. FHIR solutions are constructed from a set of modular components called “Resources” [24]. All resources have references to other resources, extensions, and human-readable extensible HTML displays.

FHIR is the evolution of interoperability standards from HL7 [14,24]. The principles of modern web and mobile development make FHIR malleable and adaptable to new technologies. Compared with other document-centric standards, HL7 FHIR takes a modular approach by exposing the health data entities as services using HTTP-based REST and API. Furthermore, FHIR is more comfortable for implementing the design of the solution for clinical and nontechnology professionals. The resources are human-readable with ﻿a modular approach, representing the atomic and granular health care data (eg, patient, procedure, medication, observation, and practitioner). FHIR’s architecture supports the inclusions of decision makers, doctors, and laboratory workers among other profiles.

The platform’s methodology considered professionals’ participation from a wide spectrum of areas in the interoperability design level (Textbox 1). They selected the data, discussed the process, and finally validated the standards adopted. HL7 FHIR helped include clinicians and laboratory decision makers in the process.

In this stage, we matched the defined minimum data set with FHIR. An FHIR-based system’s capabilities were selected by considering which resource was the most adequate from a clinical perspective. These resources can be easily assembled into working systems that solve real-world clinical and administrative problems [14]. For modeling these relations between resources, we used the clinFHIR graphBuilder application [25]. This online tool allows for the assembling of resource instances into a graph of connected resources that represent a specific scenario with FHIR.

### Software Development

The requirements from the laboratories’ providers and the Chilean government were divided into functional and nonfunctional. This phase is a critical aspect because it lays the foundation for all the software, affecting the development later in the project [26]. All functional requirements are the features the web platform will perform, such as sharing the laboratories’ tests, tracking them, and storing them safely by the Chilean government. Nonfunctional requirements describe how the web platform should behave, such as security, interoperability, and performance [27,28].

**Figure 2:**
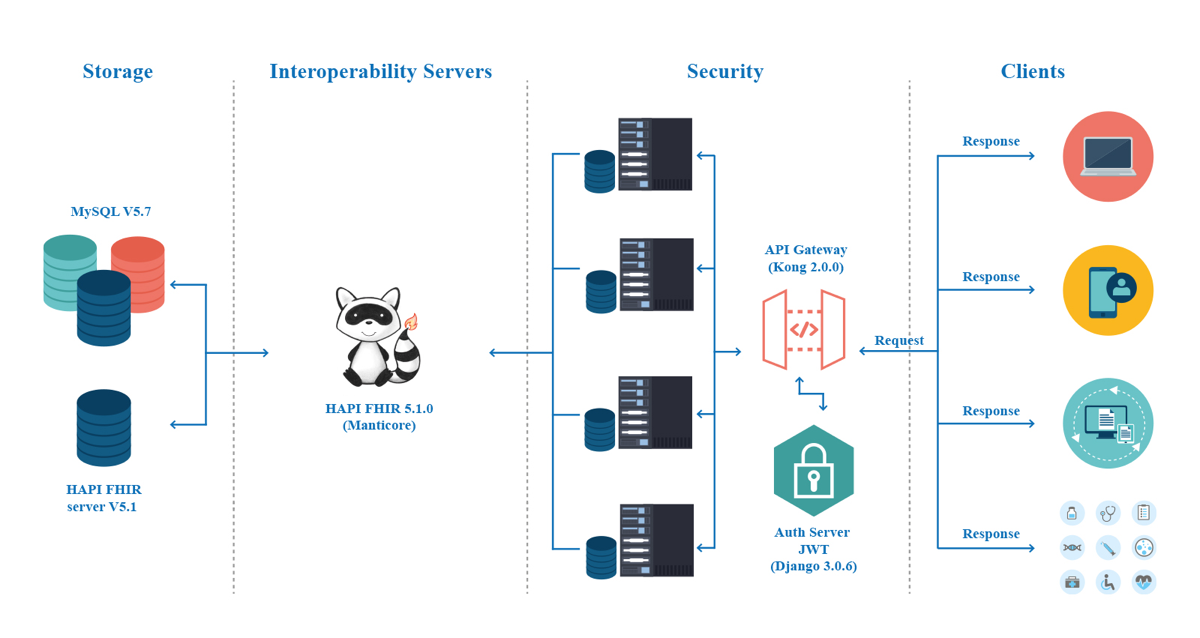
Layers of an interoperability architecture. The architecture for sharing the polymerase chain reaction SARS-CoV-2 tests from laboratories to the Chilean government. API: application programming interface; FHIR: Fast Healthcare Interoperability Resources; JWT: JavaScript Object Notation Web Token.

The software was built separating the layer from the data model and the business logic. Figure 2 shows the software architecture and technologies employed. Interoperability and security are critical nonfunctional requirements for interoperable development [14]. Moreover, the application considered a load balancer for http and https traffic, and a network load balancer for load balancing Transmission Control Protocol traffic.

The persistence (storage) was designed with two balanced mirror servers (Figure 2). This layer stored the data in a MySQL V 5.7 (Oracle Corporation) database, and the resources were stored within the HAPI FHIR database. The HAPI FHIR server V5.1 had the responsibility of the interoperability layer [18]. The HAPI FHIR library is an implementation of the FHIR specification for the Java programming language. The authentication layer was configured with JSON Web Token (JWT), an open standard [29] that defines a compact and self-contained way for securely transmitting information between parties using a JSON object.

The security infrastructure was managed by the Ministry of Health’s private network, which was outsourced to a telecommunication company. The company assumed the management and maintenance of the communications network, which includes more than 1500 health establishments throughout the country, including 120,000 voice points (telephones); 30,000 email boxes; and 200 videoconference rooms. The laboratory that participated in this platform was connected on this secure network [30].

Moreover, FHIR is suitable for use in the application layer for a broad context: mobile apps, cloud communications, electronic health care record–based data sharing, and server communication in large laboratory providers [16].

### Software Testing

Software testing was focused on functional and nonfunctional requirements. The functional requirements were obtaining the data and sharing the laboratories’ tests using FHIR, tracking PCR SARS-CoV-2 tests, and storing tests with the Chilean Ministry of Health.

**Figure 3:**
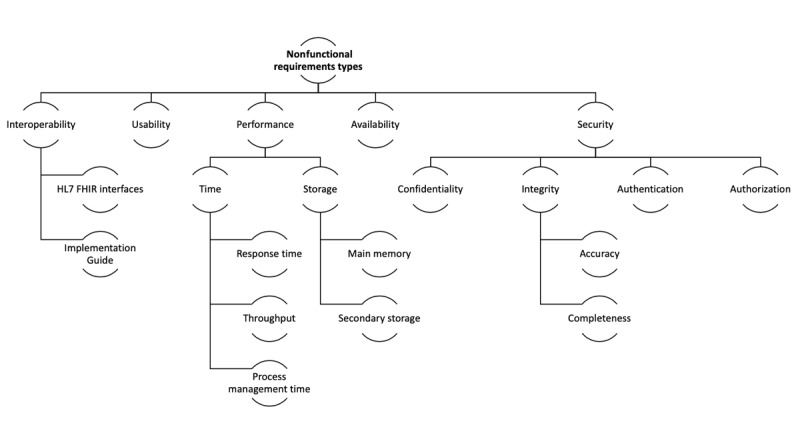
Nonfunctional requirements types for interoperable development. Catalog of nonfunctional requirements obtained from Wiegers (2005) and adapted for the interoperable platform to report polymerase chain reaction SARS-CoV-2 tests. FHIR: Fast Healthcare Interoperability Resources; HL7: Health Level 7.

The nonfunctional requirements such as performance and security are essential in health care information systems [18], such as maintaining the accuracy and completeness of the information exchanged with confidentiality and privacy. Moreover, the interoperability was the core of the solution, basing its structure on FHIR. Considering all of these issues, we proposed the catalog of nonfunctional requirements [28] by adapting the tree structure for an interoperable development (Figure 3).

### Implementation

We have implemented the platform with the UC Christus laboratory. This is primarily because they process the PCR SARS-CoV-2 tests for the “Esperanza” Project [31], a collaboration between public and private institutions to test and track COVID-19 cases in Chile.

It is critical to build a comprehensive implementation guide [14]. This guide sets rules for how an interoperability problem should be solved by employing FHIR resources. For creating the implementation guide using FHIR resources, we used the platform Simplifier.net [32].

## Results

The interoperable FHIR platform to report PCR SARS-CoV-2 tests from laboratories to the Chilean government was designed and developed following the interoperable methodology previously described. This platform could be used to report PCR SARS-CoV-2 tests from laboratories in any country with minimal changes since the interoperable methodology used is highly structured, reusable, and standardized.

### Creation of Minimum Data Set

The minimum data set was created from the stakeholder analysis and could extend its use beyond the COVID-19 pandemic. Table 1 summarizes the consented data set (see Multimedia Appendix 1).

**Table 1:** Data set of laboratory polymerase chain reaction results. The minimum data set to build the polymerase chain reaction SARS-CoV-2 report.

Field	Cardinality	Data type	Length	Description
Identification type code	1.. *	Varchar	10	Type of code that the patient used to identify themselves
Identification number	1.. *	Varchar	20	Code that identifies the patient as unique
Name	1… *	Varchar	30	Patient name
Last name	1..1	Varchar	30	Patient’s last name
Mother’s last name	1..1	Varchar	30	Patient’s mother’s name
Birth date	1..1	Date	8	Patient’s birth date in the format YYYY-MM-DD
Gender	1..1	Varchar	2	Patient’s gender
Test type	1..1	Integer	N/A^a^	Code of sample type
Test collection date	1..1	Datetime	N/A	Date and time when test collection occurred
Test reception date	1..1	Datetime	N/A	Date and time when the test was received
Laboratory code	0..1	Integer	N/A	Unique code that identifies the laboratory
Another laboratory	0..1	Text	30	When “Laboratory Code” is empty, write the laboratory’s name in this field
Test code	1..1	Varchar	10	LOINC^b^ Code identifies the test with the international terminology system [33]
Test Result	1..1	Varchar	30	LOINC identifies the result with the international terminology system [33]
Validation date	1..1	Datetime	N/A	This is when the medical technologist accepts the test result
Petition number	1..1	Integer	N/A	Value composed of 2 codes with the following format: Laboratory Code + LIS^c^ internal request code (3 + 12)

^a^N/A: not applicable.

^b^LOINC: Logical Observation Identifiers Names and Codes.

^c^LIS: Laboratory Information Systems.

### Modeling Process

**Figure 4:**
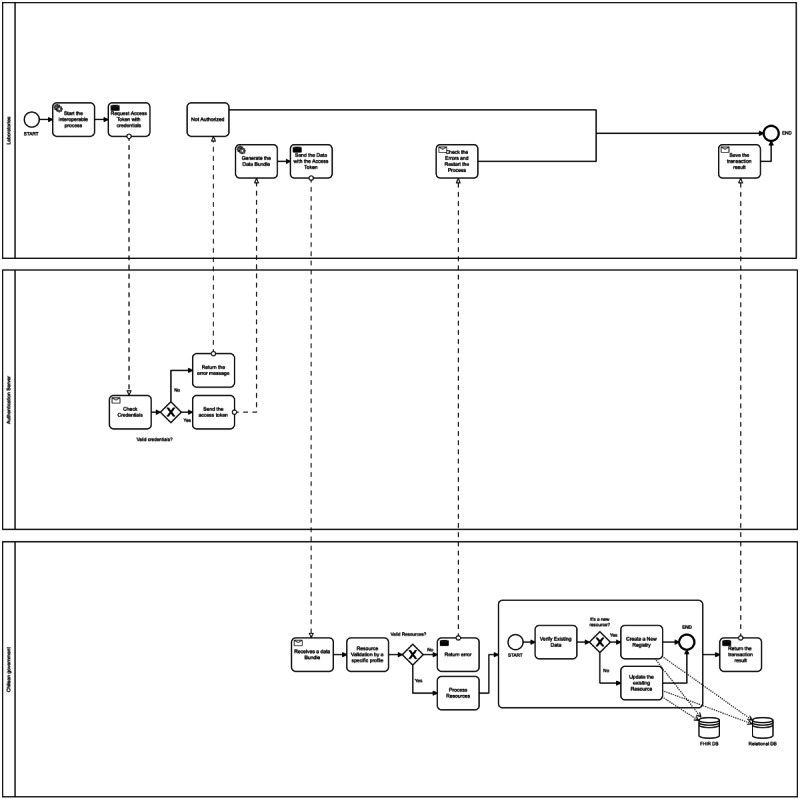
Business Process Model and Notation diagram for the interoperable platform. The process modeled defines the workflow of the information and the end points where the institutions can interchange data. DB: database; FHIR: Fast Healthcare Interoperability Resources.

The diagram created represents the complete process for obtaining the data and sharing the laboratories’ tests with FHIR, tracking PCR SARS-CoV-2 tests, and storing tests by the Chilean government. Moreover, the process identified the end point where the institutions share information (Figure 4).

### Interoperable Standards Design

The match between the minimum data set and FHIR is shown in Table 2. Each field is matched with the resources identified from FHIR. Table 2 lists the resources used for this solution.

**Figure 5:**
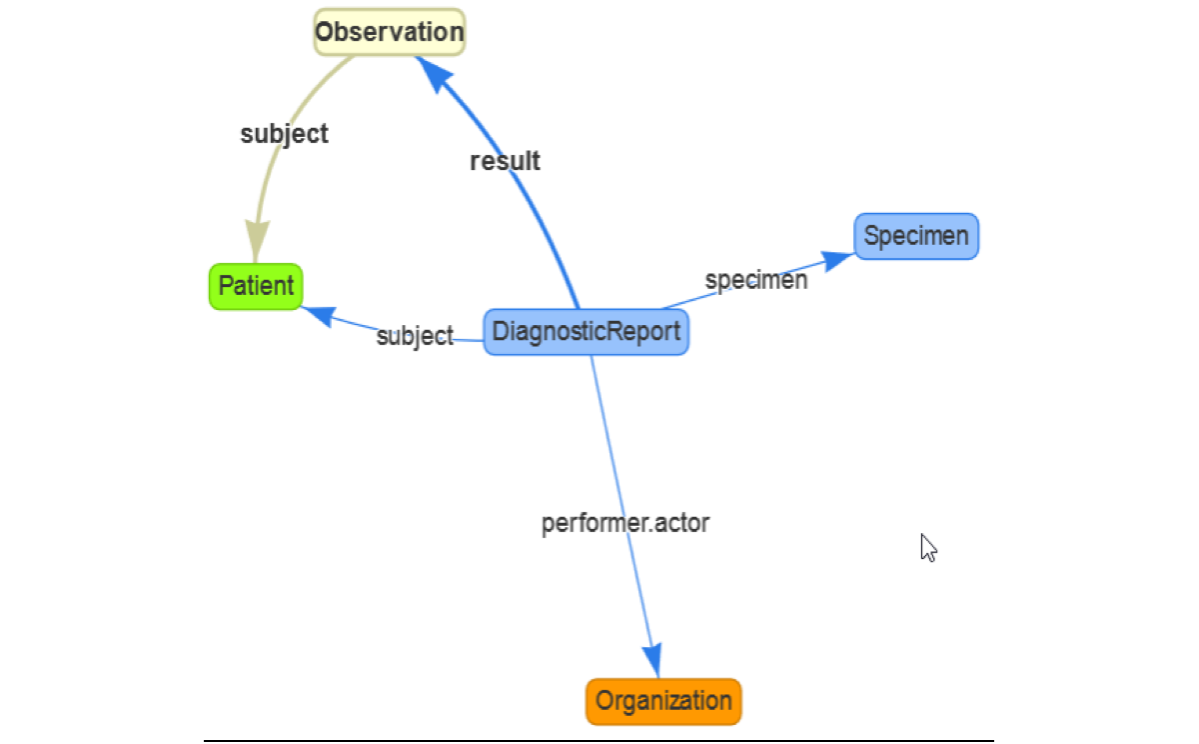
clinFHIR diagram with the resources and references. This graph shows the resources and element in the source resource that represents the reference.

With the clinFHIR modeler, we created a graph view (Figure 5). Each resource instance is represented by a rectangle containing the title and type, and references are represented by arrows (references are directional). Connecting the resources is the heart of FHIR. In this way, a complex scenario can be represented by several simple building blocks (much like the way that Lego bricks can be assembled into complex shapes). In FHIR, this process is called *references*, and a reference always goes from one resource to another.

**Table 2:** Data set of laboratory results: the minimum data set matches each element with the FHIR.

Field	FHIR^a^	Resource and element
Identification type code	Patient	Patient.identifier.type
Identification number	Patient	Patient.identifier.value
Name	Patient	Patient.name.given
Last name	Patient	Patient.name.extension
Mother’s last name	Patient	Patient.name.extension
Birth date	Patient	Patient.birthDate
Gender	Patient	Patient.gender
Test type	Specimen	Specimen.type
Test collection date	Specimen	Specimen.collection.collected
Test reception date	Specimen	Specimen.receivedTime
Laboratory code	DiagnosticReport	DiagnosticReport.performer.organization.identifier
Another laboratory	DiagnosticReport	DiagnosticReport.performer.organization.identifier
Test code	Observation	DiagnosticReport.result.code
Test result	Observation	DiagnosticReport.result.valueCodeableConcept
Validation date	Observation	DiagnosticReport.result.effectiveDateTime
Petition number	DiagnosticReport	DiagnosticReport.identifier

^a^FHIR: Fast Healthcare Interoperability Resources.

### Software Development

The agile methodology is characterized by having light and evolutionary documentation. The design documentation (BPMN process, list of requirements, match standards, database model, and sequence diagrams) is necessary to document and follow the platform’s development.

**Figure 6:**
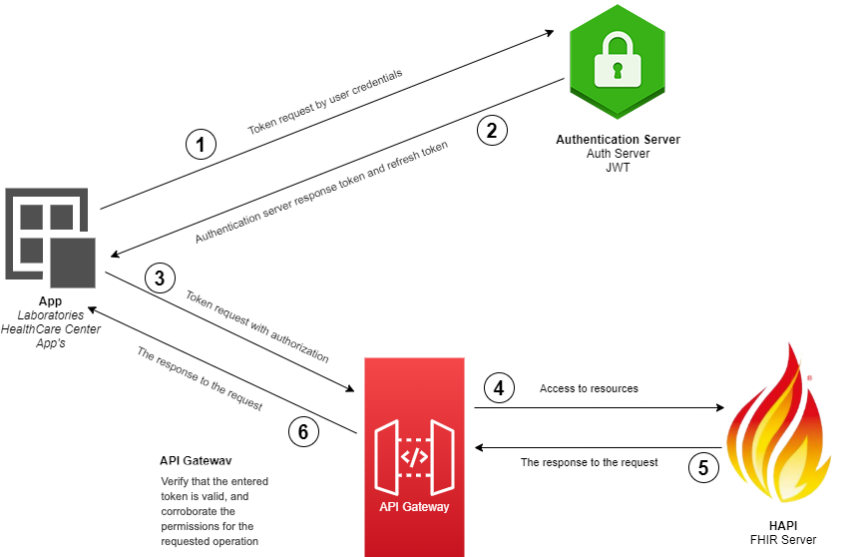
JWT authentication server. The security layer was configured with JWT to obtain valid credentials and access the HAPI FHIR server. API: application programming interface; FHIR: Fast Healthcare Interoperability Resources; JWT: JavaScript Object Notation Web Token.

The development of the proposed architecture began with the configuration of the security layer (Figure 6). This layer is responsible for managing the credentials for authorization and authentication of the valid users for accessing the HAPI FHIR server. The HAPI FHIR server was configured to develop two end points where the laboratories send the information and the Chilean government receives an FHIR message. Following this, we created profiles for each resource. An FHIR profile is a set of rules that allow an FHIR to be constrained or include extensions to add additional attributes [34].

**Figure 7:**
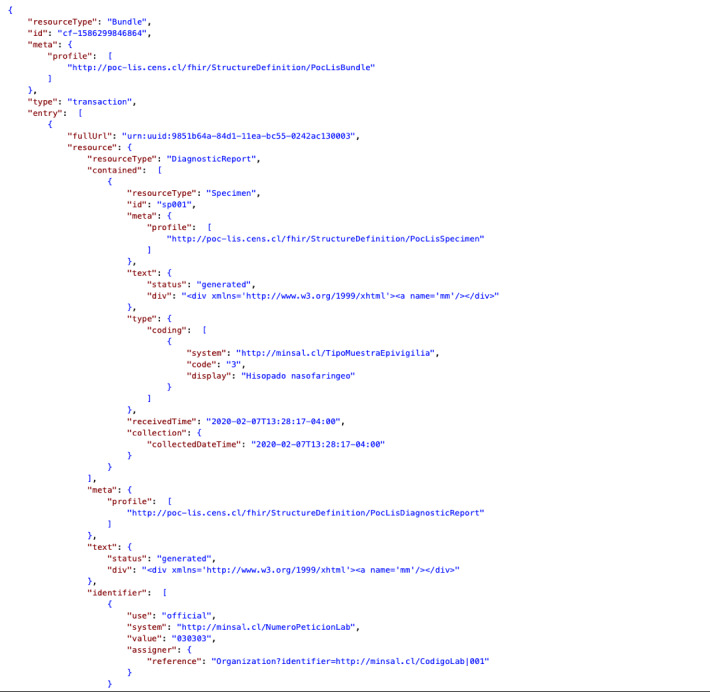
Fast Healthcare Interoperability Resources Bundle (part of the complete Bundle) that contains the resources selected for polymerase chain reaction SARS-CoV-2 tests.

At the end of this stage, we generated the message container (*Bundle*) with the list of the resources with the HAPI FHIR library (Figure 7; to review the complete *Bundle,* see Multimedia Appendix 2).

### Software Testing

Software testing was focused on testing both functional and nonfunctional requirements. The functional requirements were tested with the critical features that an interoperable web platform should have. In this sense, three functionalities were measured: (1) obtaining the data and sharing the laboratories’ tests with FHIR, (2) tracking PCR SARS-CoV-2 tests, and (3) storing tests with the Chilean government (Table 3). The FHIR and conformance statement testing used the entire bundle (Figure 7) with all the resources linked (Figure 5) with the JSON format. Each bundle sent is considered an atomic result of a PCR test belonging to one patient from UC Christus Laboratory to the Chilean government.

Nonfunctional requirements were tested considering the classification previously described (Figure 3). Table 4 lists the testing information, with the description and formulas used to calculate each result.

**Table 3:** Results from testing the functional requirements. Each functional requirement was tested.

Test	Data	Result expected	Result
Obtaining the data and sharing the laboratories’ tests with FHIR^a^	FHIR in JSON^b^ format with the full data set	Success	Bundle with links to resources created; success
Obtaining the data and sharing the laboratories’ tests with standard FHIR	FHIR in JSON format *without* full data set	Failure	Bundle with errors; failure
Tracking PCR^c^ SARS-CoV-2 tests	Request sent to FHIR server	Success	Bundle with resources; success
Storing tests with the Chilean government	Request sent with laboratory code	Success	Bundle with resources only for laboratory code; success

^a^FHIR: Fast Healthcare Interoperability Resources.

^b^JSON: JavaScript Object Notation.

^c^PCR: polymerase chain reaction.

**Table 4:** The result of testing the nonfunctional requirements. Each nonfunctional requirement was tested.

Nonfunctional requirements		Results	Description
**Interoperability**			
	HL7^a^ interfaces	FHIR^b^ R4	Interfaces with HL7 FHIR specification
	Format	JSON^c^, UML^d^, XML, and TURTLE	Several formats facilitate the use of the HL7 FHIR interfaces
	Documentation	Implementation guide based on HL7 FHIR specification	Documentation standard
Usability		Technical and nontechnical people understand FHIR resources.	The quality attribute that assesses how easy user interfaces are to use
**Performance**			
	Response time	240 milliseconds	The time they were spent waiting for a response from service: 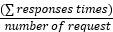
	Throughput	28.3 requests/second	Messages are processed successfully per unit of time: 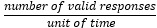
	Process management time	131 milliseconds	The time spent per task: 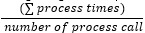
	Main memory storage	4 GB+	Main memory to store data temporarily
	Secondary storage	5.7 MB with 11,827 resources	Persistent memory to store data permanently

^a^HL7: Health Level 7.

^b^FHIR: Fast Healthcare Interoperability Resources.

^c^JSON: JavaScript Object Notation.

^d^UML: Unified Modeling Language.

A private secure network managed the security infrastructure. The laboratory was connected by a virtual private network (VPN) with the Ministry of Health. For this solution, we used the VPN for sending bundles (with all the resources involved).

For the authentication and authorization, the platform was implemented through the API gateway with valid credentials and the right access control list. This involves a set of rules or a promise usually executed through agreements that limit access or place restrictions on certain types of information. The authentication was implemented with the JWT with an expiration time, verifying the person’s or device’s identity.

The integrity was tested considering accuracy (number of mistakes that a failure detector made in a certain period) and completeness (number of crashed processes suspected by a failure detector in a certain period). We obtained (Table 4) a platform with strong accuracy [35] since each stored record could be traced back to its source and the messages were completed [35]. This is because each valid request received is processed and stored.

### Implementation

At the beginning of the implementation, a load test with 1000 requests was processed in 35 seconds. The response was under 216.7 milliseconds 50% of the time, and 90% of the time, these response times were under 323 milliseconds. The minimum and maximum response times were 118 milliseconds and 2806 milliseconds, respectively. The platform could process 28.3 requests per second. There were zero request errors. Following previous results, the solution would report 10,000 PCR SARS-CoV-2 tests in roughly 6 minutes.

The standard documentation for this solution is open for the FHIR community [15]. The implementation guide was created with Simplifier [32] for adequately documenting the scope, architecture, minimum data set, process model, profiles, and examples with the Bundle.

## Discussion

### Principal Findings

In this paper, we have designed and developed an interoperable and scalable solution that uses FHIR to access and share LIS data for reporting PCR SARS-CoV-2 tests to the Chilean government. This initiative was proposed as a use case to demonstrate the feasibility and efficiency of interoperability between heterogeneous health care information systems. The contribution was focused on supporting efficient communication in the context of the COVID-19 pandemic, collaborating with Chile’s strategy.

The WHO recommends the TTI strategy as actions that are central to containing the COVID-19 pandemic [3]. In this sense, the Chilean government has strengthened the national plan for the TTI of confirmed, suspected, and probable patients with COVID-19 and their close contacts. For this, the first two goals were the following: (1) expand the coverage of the PCR testing and bring testing closer to the community level and (2) reduce the time elapsed between detecting the positive case (by clinic or laboratory) and the epidemiological investigation [36].

To comply with the WHO’s strategy effectively and efficiently, it is essential to incorporate interoperability and information systems in the data processes involved. In this sense, we need to strengthen and make interoperable the data involved in testing and the subsequent notification to the government [37]. The LIS needs to incorporate electronic data communication via standard interfaces. This is a way to achieve efficiency and safety in laboratories that process the PCR SARS-CoV-2 tests. This means changing the current workflows that primarily require using one or more paper media, transcriptions, or copy and paste to transform raw result data into a report [9,38].

Multiple countries have had problems counting and knowing the exact number of people infected with SARS-CoV-2 [11-13]. There are various reasons and errors in the reporting of results, and a common factor is the lack of interoperability between health information systems. Approaches aimed at sending spreadsheets via email expose patients’ privacy, increase the probability of error in retyping, and generate a delay in the notification of results [11]. The involved systems lacked an interoperability strategy to address the pandemic. The data would be reported correctly if they were not retyped in different health information systems [39]. This contribution looks to alleviate health care personnel from repetitive and administrative tasks via digitalization and automation using FHIR. This reporting platform significantly streamlines sample processing and reduces turnaround time. These features are also beneficial after the initial phases of the COVID-19 crisis.

Interoperability in health care information systems has been a hard and slow process [14]. During the last decade in Chile, health information systems’ proliferation has been extensive [40]; however, each system has become a silo, cut off from other institutions [39,41]. When data must be exchanged, it is done in an ad hoc manner without standards. The COVID-19 pandemic has accelerated many digital processes incorporating essential requirements for online communication into the ecosystem. Among them is the need to interoperate between health information systems.

The interoperability area at CENS, in collaboration with Chilean laboratories (public and private), has supported a testing and traceability strategy within the “Esperanza” project [31]. In this context, CENS is promoting the development of tools and initiatives that encourage the interoperability of health information systems, making it possible to advance toward better integration and traceability for health information at the national level.

The main result of this collaboration was the development of an interoperable HL7 FHIR platform to report PCR SARS-CoV-2 tests from laboratories to the Chilean government. This contribution was centered on supporting interoperability and communication with international standards (HL7 FHIR). The described interoperable platform aims to support the efficient reporting of PCR tests with FHIR from all the national laboratories to the Chilean government. The international standards for interoperability for reporting PCR SARS-CoV-2 tests applied in our platform could be applicable and scalable in other countries, contributing to interoperability in health care information systems.

### Limitations

This platform was developed for the Esperanza COVID-19 Project [31]; however, it is a public good available to all who wish to use it.

The testing and implementation phases were applied with the back-end configuration described in the Methods section.

### Comparison With Prior Work

The prior work developed for reporting PCR SARS-CoV-2 tests from laboratories to the Chilean government was built ad hoc without standards. We made a brief comparison with the preceding solution used and found the following: the prior work does not use interoperability standards (web services–based solution), the response time of the preceding work was higher than 681 milliseconds (three times more), and a simple token managed the security without an expiration time.

### Conclusions

The FHIR platform for reporting PCR SARS-CoV-2 tests from laboratories to the Chilean government was implemented online and is currently being used with UC Christus Laboratory.

The platform was tested and implemented adequately. On average, 1000 PCR SARS-CoV-2 tests are processed in 35 seconds, with confidentiality, secure authorization and authentication, and message integrity.

This platform simplifies the reporting of PCR SARS-CoV-2 tests and contributes to reducing the time and probability of mistakes from counting positive cases.

The interoperable solution with FHIR is working successfully and is open for the community, laboratories, and any institution that needs to report PCR SARS-CoV-2 tests.

## Appendix

Multimedia Appendix 1 Minimum data set polymerase chain reaction SARS-CoV-2.

Multimedia Appendix 2 Health Level 7 Fast Healthcare Interoperability Resources Bundle polymerase chain reaction SARS-CoV-2.

## Abbreviations

API: application programming interface
BPMN: Business Process Model and Notation
CENS: National Center of Health Information Systems
FHIR: Fast Healthcare Interoperability Resources
HL7: Health Level 7
JSON: JavaScript Object Notation
JWT: JavaScript Object Notation Web Token
LIS: Laboratory Information Systems
PCR: polymerase chain reaction
REST: representational state transfer
TTI: testing, traceability, and isolation
VPN: virtual private network
WHO: World Health Organization
