# Supplementary material for: Interoperable Platform to Report Polymerase Chain Reaction SARS-CoV-2 Tests From Laboratories to the Chilean Government: Development and Implementation Study
Source: JMIR Med Inform. 2021 Jan 20;9(1):e25149. doi: 10.2196/25149 (PMC7819671; doi:10.2196/25149)
Supplement: Multimedia Appendix 1 [file medinform_v9i1e25149_app1.pdf]

Supplementary material: Minimum Dataset PCR SARS-CoV-2

<table>
<tr><th>Param</th><th>Cardinality</th><th>Data Type</th><th>Length</th><th>Description</th></tr>
<tr><td>Identification Type Code</td><td>1..*</td><td>Varchar</td><td>10</td><td>Type of code that the patient used to identify himself or herself.

Proposed codes:

<table>
<tr><th>Identification Type</th><th>Code</th></tr>
<tr><td>Birth Certificate</td><td>666784</td></tr>
<tr><td>Citizenship Card</td><td>679000</td></tr>
<tr><td>Driver License</td><td>687600</td></tr>
<tr><td>Diplomatic Passport</td><td>688000</td></tr>
<tr><td>Medical History Number</td><td>778200</td></tr>
<tr><td>Passport Number</td><td>808078</td></tr>
<tr><td>National Unique Individual Identifier</td><td>787300</td></tr>
<tr><td>Social Security Number</td><td>838300</td></tr>
<tr><td>No Personal Identification Document</td><td>836873</td></tr>
<tr><td>Unique National Provisional</td><td>787380</td></tr>
</table></td></tr>
<tr><td>Identification number</td><td>1..*</td><td>Varchar</td><td>20</td><td>Code that identifies the patient as unique.</td></tr>
<tr><td>Name</td><td>1..*</td><td>Varchar</td><td>30</td><td>Patient name.</td></tr>
<tr><td>Last Name</td><td>1..1</td><td>Varchar</td><td>30</td><td>Patient’s last name.</td></tr>
<tr><td>Mother’s Last Name</td><td>1..1</td><td></td><td>30</td><td>Patient’s mother’s name.</td></tr>
<tr><td>Birthdate</td><td>1..1</td><td>Date</td><td>8</td><td>Patient birthdate.

Format: YYYY-MM-DD</td></tr>
<tr><td>Gender</td><td>1..1</td><td>Varchar</td><td>2</td><td>Patient’s gender.

Codes:

<table>
<tr><th>Gloss</th><th>Code</th></tr>
<tr><td>Male</td><td>01</td></tr>
<tr><td>Female</td><td>02</td></tr>
<tr><td>Intersex</td><td>03</td></tr>
<tr><td>Unknown</td><td>99</td></tr>
</table></td></tr>
<tr><td>Sample Type</td><td>1..1</td><td>Integer</td><td></td><td>Code of sample type.

Codes from government’s informatics system:

<table>
<tr><th>Gloss</th><th>Code</th></tr>
<tr><td>Nasopharyngeal Swab</td><td>3</td></tr>
<tr><td>Nasopharyngeal Aspirate</td><td>4</td></tr>
<tr><td>Blood</td><td>5</td></tr>
</table></td></tr>
</table>

<table>
<tr><td></td><td></td><td></td><td></td><td><table><tr><td>Other</td><td>21</td></tr><tr><td>Bronchoalveolar Lavage</td><td>28</td></tr><tr><td>Sputum</td><td>29</td></tr><tr><td>Tracheal Aspirate</td><td>31</td></tr></table></td></tr>
<tr><td>Test Collection Date</td><td>1..1</td><td>Datetime</td><td></td><td>Date and time when test collection occurred.</td></tr>
<tr><td>Test Reception Date</td><td>1..1</td><td>Datetime</td><td></td><td>Date and time when test reception was occurred.</td></tr>
<tr><td>Laboratory Code</td><td>0..1</td><td>Integer</td><td></td><td>Unique code that identifies the laboratory</td></tr>
<tr><td>Other Laboratory</td><td>0..1</td><td>Text</td><td></td><td>When “Laboratory Code” is empty, write the laboratory’s name in this field</td></tr>
<tr><td>Test Code</td><td>1..1</td><td>Varchar</td><td>10</td><td>LOINC Code identifies the test with the international terminology system [1]

Proposed Codes:
PCR: 94309-2
Rapid IgG Test: 94507-1
Rapid IgM Test: 94508-9</td></tr>
<tr><td>Test Result</td><td>1..1</td><td>Varchar</td><td>30</td><td>LOINC (REF) Code that identify the result on international terminology system.

Accepted codes for PCR Test:
<table><tr><th>Gloss</th><th>Code</th></tr><tr><td>POSITIVE</td><td>LA11882-0</td></tr><tr><td>NEGATIVE</td><td>LA11883-8</td></tr><tr><td>INCONCLUSIVE</td><td>LA9663-1</td></tr><tr><td>UNSATISFACTORY SPECIMEN FOR EVALUATION</td><td>LA13548-5</td></tr></table>
Accepted codes for IgG and IgM:
<table><tr><th>Glosa</th><th>Código</th></tr><tr><td>POSITIVE</td><td>LA6576-8</td></tr><tr><td>NEGATIVE</td><td>LA6577-6</td></tr><tr><td>SUSPECT</td><td>LA15291-0</td></tr><tr><td>INVALID</td><td>LA15841-2</td></tr></table></td></tr>
<tr><td>Validation Date</td><td>1..1</td><td>Datetime</td><td></td><td>This is when the medical technologist accepts the test result</td></tr>
<tr><td>Petition Number</td><td>1..1</td><td>Integer</td><td></td><td>It is a value composed of 2 codes that respect the following format: Laboratory Code + LIS internal request code (3 + 12)</td></tr>
</table>

References

1. McDonald CJ, Huff SM, Suico JG, Hill G, Leavelle D, Aller R, et al. LOINC, a universal standard for identifying laboratory observations: A 5-year update. Clin Chem. 2003;49(4):624–633. PMID: 12651816
