# Supplementary material for: Interoperable Platform to Report Polymerase Chain Reaction SARS-CoV-2 Tests From Laboratories to the Chilean Government: Development and Implementation Study
Source: JMIR Med Inform. 2021 Jan 20;9(1):e25149. doi: 10.2196/25149 (PMC7819671; doi:10.2196/25149)
Supplement: Multimedia Appendix 2 [file medinform_v9i1e25149_app2.pdf]

Supplementary material: HL7 FHIR Bundle PCR SARS-CoV-2


```
{
  "resourceType": "Bundle",
  "id": "cf-1586299846864",
  "meta": {
    "profile": "http://ssms.cl/fhir/StructureDefinition/PocLisBundle"
  },
  "type": "transaction",
  "entry": [
    {
      "fullUrl": "urn:uuid:9851b64a-84d1-11ea-bc55-0242ac130003",
      "resource": {
        "resourceType": "DiagnosticReport",
        "contained": [
          {
            "resourceType": "Specimen",
            "id": "sp001",
            "meta": {
              "profile": "http://poc-lis.cens.cl/fhir/StructureDefinition/PocLisSpecimen"
            },
            "text": {
              "status": "generated",
              "div": "<div xmlns='http://www.w3.org/1999/xhtml'><a name='mm'/></div>"
            },
            "type": {
              "coding": [
                {
                  "system": "http://minsal.cl/TipoMuestraEpivigilia",
                  "code": "3",
                  "display": "Hisopado nasofaringeo"
                }
              ]
            },
            "receivedTime": "2020-02-07T13:28:17-04:00",
            "collection": {
              "collectedDateTime": "2020-02-07T13:28:17-04:00"
            }
          }
        ],
        "meta": {
          "profile": "http://poc-
lis.cens.cl/fhir/StructureDefinition/PocLisDiagnosticReport"
        },
        "text": {
          "status": "generated",
          "div": "<div xmlns='http://www.w3.org/1999/xhtml'><a name='mm'/></div>"
        },
        "identifier": [
```

```
        {
          "use": "official",
          "system": "http://minsal.cl/NumeroPeticionLab",
          "value": "12345678",
          "assigner": {
              "reference": "Organization?identifier=http://minsal.cl/CodigoLab|111111"
          }
        }
      ],
      "status": "final",
      "code": {
        "text": "Examen de Coronavirus"
      },
      "subject": {
        "reference": "urn:uuid:da824070-8445-11ea-bc55-0242ac130003"
      },
      "performer": [
        {
          "reference": "Organization?identifier=http://minsal.cl/CodigoLab|111111"
        }
      ],
      "result": [
        {
          "reference": "urn:uuid:686b2fac-93fd-11ea-bb37-0242ac130002"
        }
      ],
      "specimen": [
        {
          "reference": "#sp001"
        }
      ]
    },
    "request": {
      "method": "POST",
      "url": "DiagnosticReport"
    }
  },
  {
    "fullUrl": "urn:uuid:686b2fac-93fd-11ea-bb37-0242ac130002",
    "resource": {
      "resourceType": "Observation",
      "meta": {
        "profile": "http://poc-lis.cens.cl/fhir/StructureDefinition/PocLisObservation"
      },
      "text": {
        "status": "generated",
        "div": "<div xmlns='http://www.w3.org/1999/xhtml'>&lt;a name='mm'/&gt;</div>"
      },
      "status": "final",
      "code": {
```

```
          "coding": [
                {
                  "system": "http://loinc.org",
                  "code": "94507-1",
                  "display": "SARS coronavirus 2 Ab.IgM"
                }
          ]
        },
        "effectiveDateTime": "2020-02-07T13:28:17-04:00",
        "subject": {
          "reference": "urn:uuid:da824070-8445-11ea-bc55-0242ac130003"
        },
        "performer": [
          {
            "reference": "Organization?identifier=http://minsal.cl/CodigoLab|111111"
          }
        ],
        "valueCodeableConcept": {
          "coding": [
            {
              "system": "http://loinc.org",
              "code": "LA11882-0",
              "display": "Detected"
            }
          ]
        }
      },
      "request": {
        "method": "PUT",
        "url": "Observation?code=http://loinc.org|94507-1&value-
concept=http://loinc.org|LA11882-
0&performer.identifier=http://minsal.cl/CodigoLab|111111"
      }
    },
    {
    "fullUrl": "urn:uuid:da824070-8445-11ea-bc55-0242ac130003",
      "resource": {
        "resourceType": "Patient",
        "meta": {
          "profile": "http://poc-lis.cens.cl/fhir/StructureDefinition/PocLisPatient"
        },
        "text": {
          "status": "generated",
          "div": "<div xmlns='http://www.w3.org/1999/xhtml'><a name='mm'/></div>"
        },
        "identifier": [
          {
            "use": "official",
            "system": "http://minsal.cl/Identificacion",
            "value": "12345678",
```

```
            "extension": [
              {
                "url": "http://poc-
lis.cens.cl/fhir/StructureDefinition/TipoIdentificacionPaciente",
                "valueCoding": {
                  "system": "http://cens.cl/HCC/TipoDeIdentificacion",
                  "code": "666784",
                  "display": "Certificado de Nacimiento"
                }
              }
            ]
          }
        ],
        "name": [
          {
            "_family": {
              "extension": [
                {
                  "url": "http://poc-lis.cens.cl/fhir/StructureDefinition/humanname-
fathers-family",
                  "valueString": "Peréz"
                },
                {
                  "url": "http://poc-lis.cens.cl/fhir/StructureDefinition/humanname-
mothers-family",
                  "valueString": "Vidal"
                }
              ]
            },
            "given": "Juan Antonio",
            "use": "official",
            "text": "Juan Antonio Pérez Vidal"
          }
        ],
        "gender": "male",
        "birthDate": "1905-08-23"
      },
      "request": {
        "method": "PUT",
        "url": "Patient?identifier=http://minsal.cl/Identificacion|12345678"
      }
    }
  ]
}
```
